# Morphological Characteristics of Alveolar and Cystic Echinococcosis Lesions in Human Liver and Bone

**DOI:** 10.3390/pathogens10101326

**Published:** 2021-10-14

**Authors:** Thomas F. E. Barth, Adriano Casulli

**Affiliations:** 1 Institute of Pathology, Medical Faculty of Ulm, Ulm University, 89081 Ulm, Germany; thomas.barth@uniklinik-ulm.de; 2 WHO Collaborating Centre for the Epidemiology, Detection and Control of Cystic and Alveolar Echinococcosis, Department of Infectious Diseases, Istituto Superiore di Sanità, 00161 Rome, Italy; 3 European Union Reference Laboratory for Parasites, Department of Infectious Diseases, Istituto Superiore di Sanità, 00161 Rome, Italy

**Keywords:** Neglected Tropical Diseases, cystic and alveolar echinococcosis, *Echinococcus* spp., diagnostic pathology

## Abstract

Among echinococcoses diseases of human interest, two have a global public health impact: cystic and alveolar echinococcosis caused by *Echinococcus granulosus sensu lato* and *Echinococcus multilocularis*, respectively. Cystic and alveolar echinococcosis are neglected infectious diseases epidemiologically and are clinically vastly different with distinct microscopic features. Because of the rareness of these zoonotic diseases, pathologists have limited diagnostic experience in the analysis of the lesions caused by *Echinococcus* tapeworms. Here, we describe the main microscopic features to be considered to characterize these lesions: laminated layer, central necrosis, growth pattern, and delineation from adjacent tissue. Moreover, immunohistology using monoclonal antibodies is of great diagnostic help in reaching a definitive diagnosis by identifying the laminated body and small particles of *E. multilocularis* (spems) and small particles of *E. granulosus* (spegs).

## 1. Introduction

Echinococcoses diseases are caused by larval stages (metacestodes) of the parasitic tapeworms of the genus *Echinococcus*. In humans, alveolar echinococcosis (AE), caused by *E. multilocularis* and cystic echinococcosis (CE), induced by *E. granulosus sensu lato* (a complex of several cryptic species), are particularly important medical conditions of international public health relevance since they have a wide geographic distribution and may cause life-threatening diseases in humans [1,2]. A less frequent disease of human interest is neotropical echinococcosis (NE), caused by the tapeworm *Echinococcus vogeli*, and *E. oligarthrus*, an emerging infection in South America [3,4]. In humans the lesions of the larval stage of *Echinococcus* spp. are mainly localized in the liver. However, involvement of any tissue or organ may be possible, including the lung, lymph nodes, omentum, central nervous system, skin, and the retroperitoneum, including the paravertebral region, and bone have been described [5,6]. Because NE caused by *E. vogeli* and *E. oligarthrus* is very rare and not a well-estimated human medical condition, in the following we will mainly focus on AE and CE.

Moreover, CE and AE are among those few Neglected Tropical Diseases that are also endemic out of the tropics, including worldwide pastoral and rural communities of medium-high income countries [7]. Therefore, depending on health-care settings, these parasitic diseases should be managed as orphan or neglected infectious diseases [7,8].

Definitive diagnosis of human CE and AE is of the utmost importance since prognosis and treatment differs fundamentally [9]. The main problem for the pathologist is the rareness of this disease, resulting in an exceptionally low incidence of the parasite in the daily diagnostic routine; therefore, the pathologist generally has little diagnostic experience with this lesion outside specialized and/or experienced centers. In the following sections, we will describe the main macroscopic and microscopic features of AE and CE in humans.

## 2. Macroscopic Findings

The pathologist may come into diagnostic contact with the parasite in two ways.

**Figure 1:**
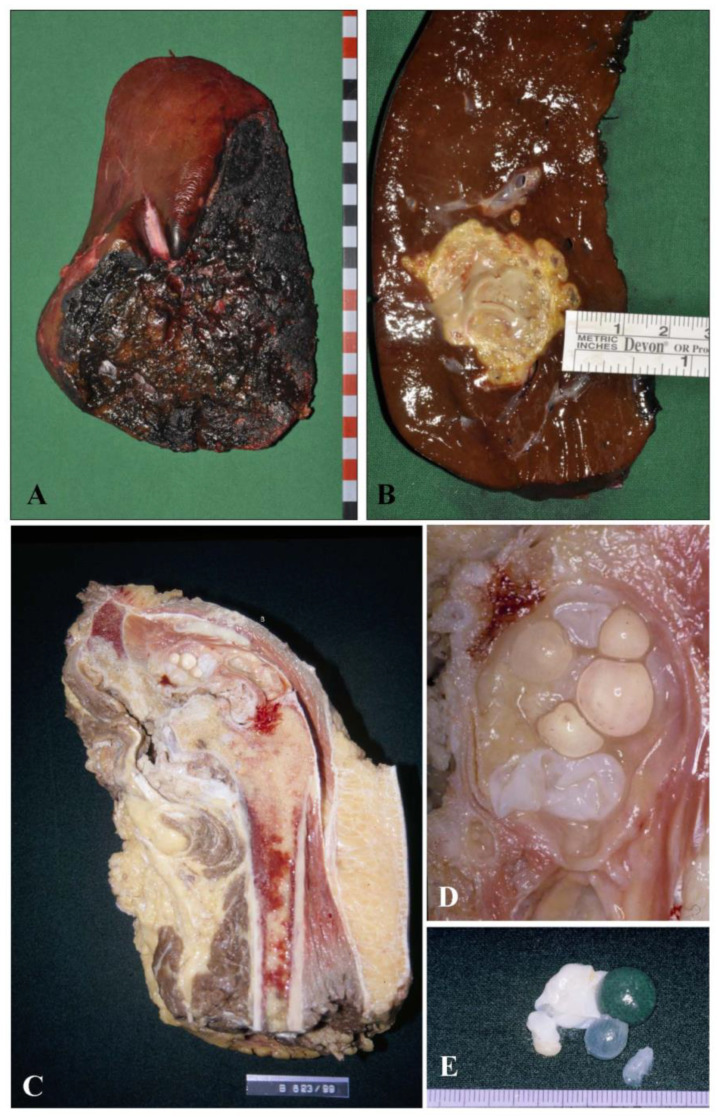
Macroscopic aspects of AE and CE infection in humans. (**A**,**B**): Hepatic manifestation of the larval stage of *E. multilocularis* in a human liver resection specimen. (**A**): The resection surface of the liver is marked with black ink; (**B**): The cutting surface shows an ill-defined, yellowish necrotic lesion; (**C**): Human hemipelvectomy specimen with destruction of the pelvis bythe larval stage of *E. granulosus*. The bone of the pelvis shows a well-demarcated lesion confined by a thick fibrotic capsule causing lyses of the pelvis; (**D**) (Higher magnification of (**C**)): Inside the lesion numerous grape-like structures are detected; (**E**): These structures are easily isolated and have a grape-like aspect with intermingled, band-like greyish formations.

1. Biopsy or cytoaspirate of an unclear lesion, such as a liver lesion of unknown dignity and entity, by means of a cutting needle biopsy or a liquid aspirate for cytological evaluation. In this instance they must be beware of some characteristic histological features of the larval stage of the parasite that will allow them to differentiate echinococcal lesion from lesions due to infectious disease, e.g., tuberculosis, fungal infections or further forms of infectious abscesses, as well as neoplastic primary or secondary metastatic lesions.
2. Description of a resection specimen of e.g., a diagnostically defined liver manifestation of the larval stage of *Echinococcus* spp., already characterized during the diagnostic workup of the bioptic material prior to elective surgery. In this instance, the pathologist must define the specimen with the lesion by measuring the lesion in centimeters and weighing the specimen. After fixation in buffered formalin (4%) for at least 24 h, the pathologist has to prepare several tissue blocks of paraffin-embedded material from the sample for documenting and archiving the lesion and defining the resection borders; they should state the distance of the lesion from the resection line (Figure 1A,B). To document clearly the distance of the lesion from the resection, we suggest marking the resection line with ink during macroscopic description of the specimen. This will allow the pathologist to precisely measure the minimal distance of the lesion from the resection line. This distance should be given in mm in the final report.

The characteristic macroscopic features may provide initial hints for the definitive diagnosis. On the cut surface in AE, there is poor delineation from surrounding tissue, the central necrosis has a yellowish color and varies from a fluid to a bread-like consistency. By contrast, the human liver lesion in CE is well delineated from surrounding tissue and generally presents as a single or multiple cysts. Within the cyst, a clear fluid with grape-like cysts is present; these may have a diameter of up to 1 cm (Figure 1C,D).

## 3. Histological Findings

**Figure 2:**
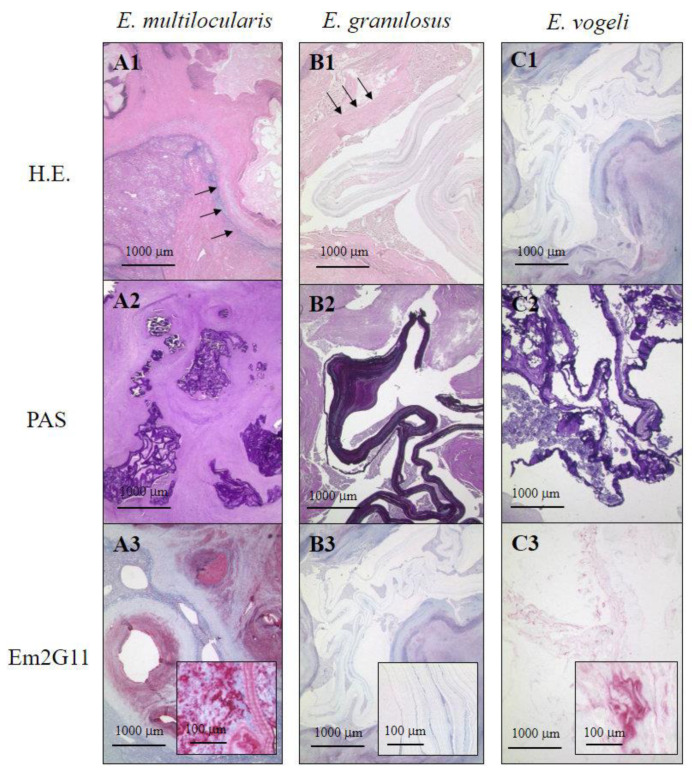
Histological aspects of infection of human hepatic tissue by *E. multilocularis, E. granulosus,* and *E. vogeli*. (**A1**–**C1**) (Hematoxylin staining). (**A1**) (*E. multilocularis*): A necrotic, poorly demarcated lesion with a chronic inflammatory infiltrate in the liver tissue is seen (see arrows); the laminated layer is very weakly stained; (**B1**) *(E. granulosus*): The laminated layer shows almost no staining, the lesion is well demarcated by a fibrotic capsule (see arrows); (**C1**) *(E. vogeli*): The laminated body shows almost no staining, the lesion is confined by a fibrotic capsule (bar = 1000 μm; equal magnification for all figures from A1 to C3). (**A2**–**C2**) (PAS staining). (**A2**) *(E. multilocularis*): The laminated layer is slender and shows a tubular growth pattern; (**B2**) *(E. granulosus*): The laminated layer is thicker and striated compared to AE and shows a deep violet PAS staining; (**C2**) (*E. vogeli*): The laminated body is of intermediate size as compared to the laminated body of AE and CE. (**A3**–**C3**) (Immunohistology with the antibody Em2G11). (**A3**): The laminated layer of *E. multilocularis* is strongly Em2G11-positive; insert shows multiple Em2G11-positive, small particles of the laminated layer of *E. multilocularis* (spems); (**B3**): The laminated layer of *E. granulosus* is negative for Em2G11 (insert with higher magnification from A3 to C3; bar = 100 μm); (**C3**): the laminated layer of *E. vogeli* shows a weak, patchy positivity for the antibody.

For a histological workup, a hematoxylin and eosin (HE) staining is mandatory as the first approach. The laminated layer of *E. multilocularis, E. granulosus,* and *E. vogeli* is hardly visible in conventional HE staining (Figure 2A1–C1). Therefore, we recommend periodic acid-Schiff (PAS) staining for every lesion that shows a central necrosis of unknown origin, since this staining allows differentiation of the lesion from a fungal infection with typical mycelium-like structures. In humans, histological detection of the laminated layer is crucial since protoscoleces and hooklets are found very rarely during histological workup, in particular with AE. The laminated layer of *E. multilocularis*, *E. granulosus,* and *E. vogeli* metacestodes mainly consists of polysaccharide protein complexes with a predominance of galactosamine over glucosamine [10]. The large amount of polysaccharides in the laminated layer is responsible for the high affinity to PAS staining in both species [11]. The laminated body has a strong positive staining in dark violet, so that the PAS staining is mandatory to allow detection of this structure.

From a histological point of view, human liver lesions of AE and CE have different aspects. In general, histology reflects the macroscopic aspect of the human liver lesion.

1. In AE the liver lesion is characterized by central necrosis of varying diameter; the necrosis may have similarities to that seen in tuberculosis; however, the area of the necrosis is generally larger in AE and the typical multinuclear giant of the tuberculosis infection occurs only rarely or not at all. Calcifications may be present within the necrosis; these calcifications are generally not solid but have the characteristics of an impregnation with a dot-like or granular pattern. Ossifications are not detected. Next follows an inner circle close to the necrotic zone characterized by epithelioid cells, macrophages, and granulocytes; the granulocytes are predominantly of the neutrophilic type; some eosinophilic granulocytes may be present as well as some giant cells. This is followed by an outer zone with numerous lymphocytes followed by hepatic tissue. Between the outer and inner a fibrotic layer of varying diameter is present.
2. In CE there is no central necrosis; the lesion is characterized by a broad fibrotic capsule with a limited lymphocytic infiltrate around the fibrotic capsule.

A central microscopic feature of AE and CE is the detection and description of the laminated layer. As outlined above, the laminated layer is strongly PAS-positive. Comparing the shape of the laminated layer reveals important differences, allowing AE to be differentiated from CE. In AE the laminated layer is recognized as small PAS-positive particles with a slender shape and a bizarre configuration with a maximum size of 1 mm (Figure 2A2). The PAS staining shows that the lesion is poorly confined to the surrounding tissue and tubular structures expand in the surrounding liver tissue. Of note, fragments of protoscoleces are found only very rarely in human tissue, in contrast to liver tissue of the mouse, reflecting a different type of efficiency of inflammation in mice and humans. Therefore, the histological hallmarks in both lesions are the PAS-positive remnants of the laminated layer.

By contrast, in CE, at the histological level, the larval stage is characterized by a broad fibrotic rim that includes the echinococcal cyst. The laminated layer is only weakly positive in HE staining, therefore, a PAS staining should be performed (Figure 2B1,B2). The PAS staining shows a completely different arrangement of the laminated body compared to AE. In the PAS staining, the laminated layer is strongly positive and has a violet staining, which is even darker in color with striation when compared to AE and has been compared to a vinyl disk; the laminated body is much broader than the laminated layer of AE and measures up to 3 mm. A tubular growth pattern is not present in the liver, reflecting the macroscopic aspect of the lesion. Within the lesion, protoscoleces or scolex remnants are very rarely detected in AE lesions. Therefore, the pathologist has to focus all their attention on the laminated layer. The major macroscopic and microscopic differences between AE and CE in infection in humans are summarized in a list in Table 1.

By considering the above-mentioned differences (shape of the laminated layer, absence versus presence of necrosis, delineation from adjacent tissue) a histological diagnosis is possible in more than 90% of cases [5,12]. Difficulties may arise e.g., in aspirates with only small amounts of diagnostic material.

**Figure 3:**
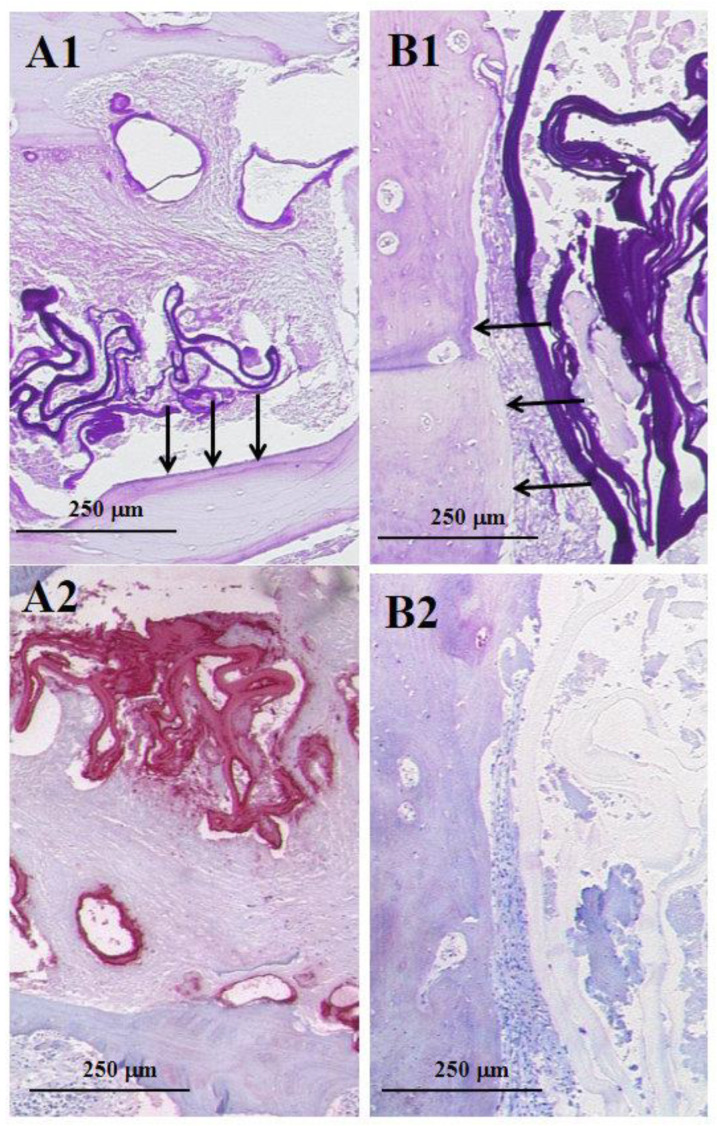
Spectrum of bone lesions of AE and CE. In a PAS staining, the laminated layer of AE and CE is slender in both lesions and may therefore be confused. Arrows mark trabecular bone; (**A1**,**B1**). Staining with the antibody Em2G11 shows a strong positive staining for AE, whereas CE is negative (**A2**,**B2**); all figures (from A1 to B2) have equal magnification; bar = 250 μm).

Manifestations of AE or CE in the bones may be difficult and cause uncertainties if the above-mentioned parameters are used, since the laminated layer in bone lesions of CE may be as slender as in AE (Figure 3A1,A2,B1,B2). In such cases, the pathologist must integrate all the available clinical data, including serum markers and radiographic findings, when making the diagnosis.

## 4. Immunohistological Findings

A major diagnostic breakthrough has been achieved with the monoclonal antibody Em2G11, which is highly specific for the laminated layer of *E. multilocularis*. The antigen recognized by the antibody is the mucin-type Em2 in the laminated layer of the *E. multilocularis* metacestodes.

It is suggested that, due to this antigen, the metacestode of AE escapes the host immune response as shown in animal models [13,14,15] by modulating the T-cell response and activating a T-cell-independent B-cell reaction, which lacks antibody maturation. With conventional immunohistochemical staining of paraffin sections of archived paraffin material, staining with the antibody is highly sensitive and specific for AE [16]. Furthermore, this technique revealed an unknown feature of human AE that we called “small particles of *E. multilocularis*”, i.e., spems [5,17,18]. We further have described the presence of these particles in CE and called these (in analogy to spems) small particles of. *E. granulosus* (spegs) by the use with monoclonal antibody EmG3 directed against an antigen, which has not been characterized yet [12]. We argue that these small particles represent micro-fragments of *E. multilocularis* and *E. granulosus* since spems and spegs are also found in sinusoids of the liver, as well as in draining lymph nodes around the main lesion. Therefore, the potential interface of interaction of the parasite and the host is much larger than initially assumed and thus points to a further form of host–parasite interaction. These particles seem not be infective and the presence of spems in lymphnodes does not seem to be associated with recurrent disease, although these lymphnodes are enlarged [18,19]. On the basis of these morphological and immunohistological findings, we developed a diagnostic algorithm for the differential diagnosis of echinococcosis [12]. We had the opportunity to analyze one of the rare cases of a human infection of *E. vogeli* (Figure 2(C1–C3)). The laminated layer is smaller than is seen in AE and larger than the laminated layer of AE. Staining with the antibody Em2G11 shows a weak, patchy pattern. We concluded that, from a morphological point of view, *E. vogeli* has features overlapping with AE and CE and resembles both of them [20]. Regarding this finding, however, more cases have to be analyzed in larger studies.

## 5. Conclusions

AE and CE have some distinct macroscopic and microscopic features. A necrotic and poorly confined lesion characterizes the macroscopic feature of AE in the human liver; histology shows a typical tubular growth pattern. By contrast, the lesion of CE is generally well circumscribed on macroscopic grounds; histology reveals a broad laminated layer. A PAS staining is mandatory for precise diagnosis and the differences in the shape of the laminated layer are the diagnostic criteria (slender in AE and thick in CE). Discrimination is difficult with aspirates or in bone lesions of AE and CE. Immunochemical staining using the AE-specific monoclonal antibody Em2G11 and monoclonal antibody EmG3 are of great diagnostic help in reaching a definitive diagnosis of AE and CE by identifying the laminated body and small particles of *E. multilocularis* (spems) and small particles of *E. granulosus* (spegs) even on cytological aspirates [5].

## Figures and Tables

**Table 1:** Conventional macroscopic and histological diagnostic criteria for the differential diagnosis of alveolar (AE) and cystic echinococcosis (CE).

Criteria	AE	CE
**Macroscopy**	Multiple Small Vesicles	Generally Single but Larger Cyst (or Multiple Cysts)
**Microscopic features**		
Laminated layer (PAS staining)	Slender (below 1 mm) with weak striation	Thick (up to 3 mm) with strong to moderate striation
Central necrosis	Abundant	Little or none
Growth pattern	Tubular	Pseudocystic
Delineation from adjacent tissue	Poorly delineated	Pseudocystic lesion with thick fibrous capsule

## Data Availability

Data available on request.

## References

[1] Eckert J., Deplazes P., Kern P., Brown D., Palmer S., Torgerson P.R., Soulsby E. (2011). Alveolar echinococcosis (*Echinococcus multilocularis*) and other forms of echinococcosis (*Echinococcus vogeli* and *Echinococcus oligarthrus*). Zoonoses.

[2] Casulli A. (2020). Recognising the substantial burden of neglected pandemics cystic and alveolar echinococcosis. Lancet Glob. Health.

[3] D’Alessandro A., Rausch R.L. (2008). New aspects of neotropical polycystic (*Echinococcus vogeli*) and unicystic (*Echinococcus oligarthrus*) echinococcosis. Clin. Microbiol. Rev..

[4] Vuitton D.A., McManus D.P., Rogan M.T., Romig T., Gottstein B., Naidich A., Tuxun T., Wen H., Menezes da Silva A. (2020). World Association of Echinococcosis. International consensus on terminology to be used in the field of echinococcoses. Parasite.

[5] Barth T.F.E., Herrmann T.S., Tappe D., Stark L., Grüner B., Buttenschoen K., Hillenbrand A., Juchems M., Henne-Bruns D., Kern P. (2012). Sensitive and specific immunohistochemical diagnosis of human alveolar echinococcosis with the monoclonal antibody Em2G11. PLoS Negl. Trop. Dis..

[6] Buttenschoen K., Kern P., Reuter S., Barth T.F. (2009). Hepatic infestation of *Echinococcus multilocularis* with extension to regional lymph nodes. Langenbeck’s Arch. Surg..

[7] Casulli A. (2021). New global targets for NTDs in the WHO roadmap 2021–2030. PLoS Negl. Trop. Dis..

[8] Junghanss T., da Silva A.M., Horton J., Chiodini P.L., Brunetti E. (2008). Clinical management of cystic echinococcosis: State of the art, problems, and perspectives. Am. J. Trop. Med. Hyg..

[9] Brunetti E., Kern P., Vuitton D.A. (2010). Writing Panel for the WHO-IWGE. Expert consensus for the diagnosis and treatment of cystic and alveolar echinococcosis in humans. Acta Trop..

[10] Díaz A., Casaravilla C., Irigoín F., Lin G., Previato J.O., Ferreira F. (2011). Understanding the laminated layer of larval *Echinococcus* I: Structure. Trends Parasitol..

[11] Thompson R.C.A., Thompson R.C.A. (1995). Biology and systematics of *Echinococcus*. Echinococcus and Hydatid Disease.

[12] Reinehr M., Micheloud C., Grimm F., Kronenberg P.A., Grimm J., Beck A., Nell J., Meyer Zu Schwabedissen C., Furrer E., Müllhaupt B. (2020). Pathology of Echinococcosis: A Morphologic and Immunohistochemical Study on 138 Specimens with Focus on the Differential Diagnosis Between Cystic and Alveolar Echinococcosis. Am. J. Surg. Pathol..

[13] Deplazes P., Gottstein B. (1991). A monoclonal antibody against *Echinococcus multilocularis* Em2 antigen. Parasitology.

[14] Hülsmeier A.J., Gehrig P.M., Geyer R., Sack R., Gottstein B., Deplazes P., Köhler P. (2002). A major *Echinococcus multilocularis* antigen is a mucin-type glycoprotein. J. Biol. Chem..

[15] Gottstein B., Soboslay P., Ortona E., Wang J., Siracusano A., Vuitton D.A. (2017). Immunology of Alveolar and Cystic Echinococcosis (AE and CE). Adv. Parasitol..

[16] Dezsényi B., Strausz T., Makrai Z., Csomor J., Danka J., Kern P., Rezza G., Barth T.F., Casulli A. (2017). Autochthonous human alveolar echinococcosis in a Hungarian patient. Infection.

[17] Ricken F.J., Nell J., Grüner B., Schmidberger J., Kaltenbach T., Kratzer W., Hillenbrand A., Henne-Bruns D., Deplazes P., Moller P. (2017). Albendazole increases the inflammatory response and the amount of Em2-positive small particles of *Echinococcus multilocularis* (spems) in human hepatic alveolar echinococcosis lesions. PLoS Negl. Trop. Dis..

[18] Grimm J., Nell J., Hillenbrand A., Henne-Bruns D., Schmidberger J., Kratzer W., Gruener B., Graeter T., Reinehr M., Weber A. (2020). Immunohistological detection of small particles of *Echinococcus multilocularis* and *Echinococcus granulosus* in lymph nodes is associated with enlarged lymph nodes in alveolar and cystic echinococcosis. PLoS Negl. Trop. Dis..

[19] Hillenbrand A., Beck A., Kratzer W., Graeter T., Barth T.F.E., Schmidberger J., Möller P., Henne-Bruns D., Gruener B. (2018). Impact of affected lymph nodes on long-term outcome after surgical therapy of alveolar echinococcosis. Langenbeck’s Arch. Surg..

[20] Stijnis K., Dijkmans A.C., Bart A., Brosens L.A., Muntau B., Schoen C., Barth T.F., van Gulik T., van Gool T., Grobusch M.P. (2015). *Echinococcus vogeli* in immigrant from Suriname to the Netherlands. Emerg. Infect. Dis..

